# An improved genome release (version Mt4.0) for the model legume Medicago truncatula

**DOI:** 10.1186/1471-2164-15-312

**Published:** 2014-04-27

**Authors:** Haibao Tang, Vivek Krishnakumar, Shelby Bidwell, Benjamin Rosen, Agnes Chan, Shiguo Zhou, Laurent Gentzbittel, Kevin L Childs, Mark Yandell, Heidrun Gundlach, Klaus FX Mayer, David C Schwartz, Christopher D Town

**Affiliations:** 1 J. Craig Venter Institute, 9704 Medical Center Drive, Rockville, MD, USA; 2 Laboratory for Molecular and Computational Genomic, Department of Chemistry, University of Wisconsin-Madison, Madison, WI, USA; 3 Université de Toulouse, INP-ENSAT, CNRS, Laboratoire d’Écologie Fonctionnelle et Environnement, Toulouse, France; 4 Department of Plant Biology, Michigan State University, East Lansing, MI, USA; 5 Department of Human Genetics, University of Utah, Salt Lake City, Utah, USA; 6 MIPS/IBIS Inst. for Bioinformatics and System Biology, Helmholtz Center Munich, German Research Center for Environmental Health (GmbH), Neuherberg, Germary

**Keywords:** Medicago, Legume, Genome assembly, Gene annotation, Optical map

## Abstract

**Background:**

*Medicago truncatula*, a close relative of alfalfa, is a preeminent model for studying nitrogen fixation, symbiosis, and legume genomics. The Medicago sequencing project began in 2003 with the goal to decipher sequences originated from the euchromatic portion of the genome. The initial sequencing approach was based on a BAC tiling path, culminating in a BAC-based assembly (Mt3.5) as well as an in-depth analysis of the genome published in 2011.

**Results:**

Here we describe a further improved and refined version of the *M. truncatula* genome (Mt4.0) based on *de novo* whole genome shotgun assembly of a majority of Illumina and 454 reads using ALLPATHS-LG. The ALLPATHS-LG scaffolds were anchored onto the pseudomolecules on the basis of alignments to both the optical map and the genotyping-by-sequencing (GBS) map. The Mt4.0 pseudomolecules encompass ~360 Mb of actual sequences spanning 390 Mb of which ~330 Mb align perfectly with the optical map, presenting a drastic improvement over the BAC-based Mt3.5 which only contained 70% sequences (~250 Mb) of the current version. Most of the sequences and genes that previously resided on the unanchored portion of Mt3.5 have now been incorporated into the Mt4.0 pseudomolecules, with the exception of ~28 Mb of unplaced sequences. With regard to gene annotation, the genome has been re-annotated through our gene prediction pipeline, which integrates EST, RNA-seq, protein and gene prediction evidences. A total of 50,894 genes (31,661 high confidence and 19,233 low confidence) are included in Mt4.0 which overlapped with ~82% of the gene loci annotated in Mt3.5. Of the remaining genes, 14% of the Mt3.5 genes have been deprecated to an “unsupported” status and 4% are absent from the Mt4.0 predictions.

**Conclusions:**

Mt4.0 and its associated resources, such as genome browsers, BLAST-able datasets and gene information pages, can be found on the JCVI Medicago web site (http://www.jcvi.org/medicago). The assembly and annotation has been deposited in GenBank (BioProject: PRJNA10791). The heavily curated chromosomal sequences and associated gene models of Medicago will serve as a better reference for legume biology and comparative genomics.

## Background

Legumes contribute a significant portion of protein and oil intake in human and animal diets. An agronomically significant feature of the legume plants is their ability to fix atmospheric nitrogen as a result of symbiosis with rhizobial bacteria. Among the legumes, *Medicago truncatula* naturally stands out as a model legume organism, with several unique characteristics: compact genome size (estimated ~465 Mb according to Plant C-values database [http://data.kew.org/cvalues/] [1]), rapid life cycle, accessible genetics tools including transposon tagging and easy transformation, as well as a rich collection of mutants and ecotypes. Research on Medicago has focused on symbiotic nitrogen fixation [2] as well as a reference for cross-legume comparisons. A high-quality *M. truncatula* reference genome and gene models provide a solid foundation for plant physiologists and legume biologists, therefore, deserve continuous improvement.

The *M. truncatula* sequencing project began in 2003 with the National Science Foundation (NSF) and the European Union’s Sixth Framework Program providing initial funding to complete sequencing of the euchromatic portion of the genome, which was first estimated to be ~40% of the genome but later re-adjusted to ~280-300 Mb, necessitating a second round of NSF funding. Among the eight chromosomes in Medicago, six were sequenced in the US by the NSF-funded projects, one (chromosome 5) was sequenced by Genoscope in France with funding from the European Union and Institute for Agricultural Research (INRA), and one (chromosome 3) was sequenced in the United Kingdom with funding from the European Union and Biotechnology and Biological Sciences Research Council (BBSRC). Subsequent to the completion of the BAC-based assembly phase, ongoing efforts (in collaboration with the Medicago HapMap project) are aimed at completing the genome and its gene inventory using Next Generation sequencing methods.

Mt3.5 was mostly Sanger-based, with chromosomes built using overlapping BACs that were assembled to a total of ~250 Mb sequences, representing most of the euchromatic space. The remaining sequences were mostly short contigs derived from Illumina sequencing and amounted to an additional ~100 Mb of sequence. Genome annotation was carried out by the International Medicago Genome Annotation Group (IMGAG; http://medicago.org/genome/IMGAG/), generating a uniform set of annotations of the gene-rich pseudomolecules, the unanchored BACs, and the Illumina assemblies not captured by the BAC-based assemblies. A detailed analysis on Mt3.5 was published in 2011 [3].

Following the release of Mt3.5 and catalyzed by the plummeting sequencing costs, we embarked upon a new whole genome shotgun sequencing using Illumina technology to produce a more complete and accurate assembly of the entire genome. The final product is a hybrid, whose backbone is a *de novo* assembly of whole genome shotgun (WGS) sequences, and enhanced where appropriate with high quality BAC sequences from the Mt3.5 assembly. In addition to the paired ends and mate pairs of DNA fragments, optical and genetic map data have been used to validate and guide the long-range assembly of chromosomes [4,5]. The eight pseudomolecules now span ~384 Mb (of which ~366 Mb is actual sequence). Another ~28 Mb of sequence is found in scaffolds that cannot be anchored to either the physical or genetic maps, representing an overall anchoring rate of ~93%. Almost all of the sequences that previously resided on short Illumina contigs in the Mt3.5 release have now been incorporated into the pseudomolecules.

With the new assembly, a new annotation release becomes necessary. Even when the underlying sequences have not been updated, more transcriptional and translational evidence as well as new *ab initio* prediction methods can improve the annotation of a genome [6,7]. In Mt4.0, we have re-annotated the Medicago gene structures using a hybrid pipeline intended to both preserve well-supported gene structures from Mt3.5, and also to improve, extend or instantiate novel structures. In essence, the current set of gene models is a union of genes predicted by Evidence Modeler (EVM) [8] and MAKER [9], supplemented with custom curated gene sets provided by collaborators. We have also binned the gene predictions into two sets: high confidence and low confidence, and have flagged loci that appear to be transposable element (TE)-related. Our annotation release contains a set of genes that have retained their overall structures and identifiers, as well as a set of genes that are mostly derived from the new sequences added to the chromosomes that previously resided on unanchored BACs or short Illumina contigs. The Mt4.0 release, including the assembly and the annotation, has been released to Genbank and the JCVI Medicago website. The JCVI Medicago website also features a number of tools to facilitate queries and navigation of the Mt4.0 genomic datasets.

## Methods

### Genome assembly overview

**Figure 1:**
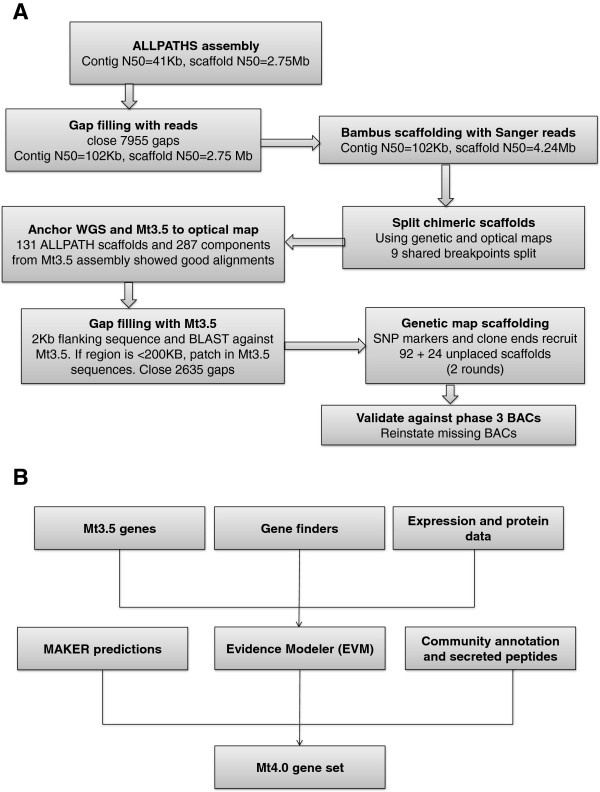
Overview of (A) assembly and (B) annotation strategies used in the Mt4.0 genome release.

The new Medicago Mt4.0 assembly is largely based on an ALLPATHS-LG [10] assembly using a combination of sequence types as described below. The ALLPATHS scaffolds were then ordered and oriented to build the pseudomolecules based on optical map, genetic map and BAC/fosmid-end sequences. Scaffolding gaps and sequencing gaps were patched and closed by the Mt3.5 assembly when possible. The entire assembly pipeline can be viewed in Figure 1A, with key steps detailed below in order.

### De novo assembly

ALLPATHS-LG (version R41245) was run with default settings. The reads used as input to the ALLPATHS-LG assembler are shown in Table 1. The set of input reads contain a mixture of sequencing technologies including Illumina, 454 and Sanger. Illumina sequencing comprise the bulk of the sequencing depth, with ~90X short fragments (paired-end) and ~50X long jumps (mate-pairs). Sanger-sequenced BAC/fosmid-ends were trimmed to use base positions between 100-250 bp prior to assembly. Following the ALLPATHS-LG assembly, we performed gap closure using GapCloser [11] at K-mer setting of 31. We performed further scaffolding using Sanger “long-jump” reads (BAC/fosmid-end sequences), which were likely under-used by ALLPATHS-LG. To perform scaffolding with the BAC/fosmid ends, BLAST was used to map the paired reads to the assembly (≥95% identity, ≥ 100 bp alignment) to provide input to the standalone scaffolder, Bambus [12]. Bambus required a minimum of 3 links to join contigs or scaffolds.

**Table 1:** Summary of sequencing libraries as input to the ALLPATHS-LG assembler

**Type**	**Library name**	**Library size**	**# of reads**	**Sequence coverage (X-fold)**	**# of pairs**	**Pair coverage (X)**
Frag	Illumina PE-200	207 ± 40	212,635,636	49.5	84,508,836	57.9
Frag	Illumina PE-376	244 ± 75	269,583,440	48.3	87,087,424	73.1
Frag	total		482,219,076	97.8	171,596,260	131.1
Jump	Illumina 3Kb	2014 ± 785	117,669,776	13.2	19,112,272	34
Jump	Illumina 4.5Kb	4866 ± 549	78,918,228	7.3	6,426,863	104.2
Jump	Illumina 5Kb	5062 ± 776	200,273,082	32.5	9,097,003	154.9
Jump	Illumina 7Kb	7455 ± 998	50,076,448	0.8	424,740	10.8
Jump	454 FLX 3Kb	2260 ± 816	1,499,510	0.4	440,037	3.7
Jump	total		448,437,044	54.2	35,500,915	307.7
Long_jump	Fosmid lib	35000 ± 7000	68,372	0	7,626	0.8
Long_jump	BAC lib mtrs	65000 ± 13000	40,080	0	9,269	1.9
Long_jump	BAC libs mte1 and mth2	100000 ± 20000	151,538	0	38,306	14.5
Long_jump	BAC lib mth4	200000 ± 40000	17,042	0	4,303	2.7
Long_jump	Total		277,032	0.1	59,504	19.9

Type refers to the ALLPATHS terminology of sequencing libraries - “frag” refers to short insert paired-end libraries that are typically two ends of <1Kb fragments, “jump” refers to long insert mate pair libraries that are typically between 1Kb to 10Kb, “long_jump” refers to the ends of fosmids and BACs.

### Construction of high density linkage map

Individuals from a Medicago recombinant inbred line [RIL; DZA315.16 x Jemalong.J6 [13]] mapping population were used to generate a high density genetic map using the GBS (genotyping-by-sequencing) method [14]. DNA from 141 individuals (among which two individuals were the parental accessions) were digested with the ApeKI restriction enzyme and ligated to Illumina single-end adapters and barcodes. Following sequencing, GBS reads were deconvoluted and mapped to the ALLPATHS scaffolds using BWA [15] using only uniquely mapped reads. SNPs were called using SAMtools mpileup [16]. The genotype at each SNP locus was labeled either as ‘A’ (same as reference allele) or ‘B’ (alternative allele) for each individual, if the allele was supported by at least 3 reads. We labeled the genotype as ‘-’ (missing data) if multiple alleles were found (i.e. heterozygous). A SNP marker was considered as ‘segregating’ if the minor allele had a frequency of at least 0.1 (this low threshold was applied in order to include markers on chr3L, most of which showed severe segregation distortion). We further required each marker to contain no more than 25% of missing data across 139 individuals to discard non-informative markers. In summary, the GBS map contains 12,002 SNP markers for 139 mapping individuals. A total of 285 SSR markers had already been mapped on this set of LR4 RILs [13] and were consolidated with the GBS map, providing an integrated map with a combined marker number of 12,287.

### Anchor WGS scaffolds

**Figure 2:**
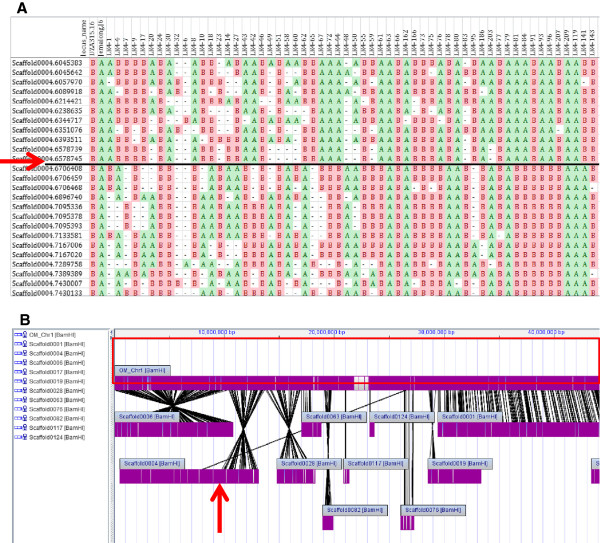
**Example of breakpoint identification using (A) GBS map and (B) optical map alignment.** Red arrows indicate the same breakpoint on Scaffold0004 indicated by GBS map and optical map alignment.

Prior to constructing the pseudomolecules, chimeric WGS scaffolds were split using the GBS map and optical map alignments. First, the segregation patterns between adjacent GBS markers were compared and a flag was raised when adjacent segregation patterns differed more than 10% (14) of the mapping individuals. Using the GBS map, we identified a total of 26 breakpoints. The optical map alignments using established methods [17-19] suggested 11 breakpoints within chimeric scaffolds (defined as scaffolds aligning to two different chromosomes in the optical map), among which 9 were shared with the genetic map breakpoints. The WGS scaffolds were split at the boundaries of these 9 identified chimeric positions that were supported by both the GBS and the optical map (Figure 2).

Alignments of the WGS scaffolds to the optical map ordered and oriented them for tiling the ALLPATHS scaffolds. A total of 131 ALLPATHS scaffolds could be anchored to the chromosomal optical maps to form preliminary pseudomolecules. However, some regions in the optical map were not yet covered by the new sequence assemblies, but had good sequence matches from the Mt3.5 assemblies, suggesting that these sequences can be recruited into Mt4.0 in a mix-and-match fashion. A total of 287 sequence segments from the Mt3.5 assembly that showed good alignments to optical map and fell in the gaps of the new assembly were inserted into the new assembly.

### Additional round of gap filling and sequence anchoring

The optical map alignment identified all the large components that were not yet placed on the pseudomolecules. There were additional sequence gaps, mostly within scaffolds, in the new assembly that could be patched using the sequences from the Mt3.5 assembly. Sequences flanking each gap in the provisional Mt4.0 assembly (2Kb on each side) were extracted and searched against the Mt3.5 assembly (BLAST; ≥ 99% identity, word size = 100). A gap was considered as closeable if sequences from both sides of the Mt4.0 gap mapped within 200Kb and with the same orientation on the Mt3.5 assembly and if the new patch sequence contained fewer N’s than the original gap. A total of 2,653 gaps (out of 8,292 examined) were closed using this process.

Some unplaced scaffolds that did not have obvious optical map alignments still contained SNP markers or clone ends that were linked to the reconstructed pseudomolecules. We anchored these scaffolds if their placements were supported by both genetic map (at least 1 linked SNP marker) and clone ends mappings (at least 1 clone end link). We performed two rounds of such scaffolding. The first round recruited 92 unplaced scaffolds and the second round recruited 24 unplaced scaffolds. Together, these steps recruited additional sequences spanning ~26.5 Mb on the pseudomolecules.

We further evaluated WGS scaffolds against high quality sequence components from Mt3.5 assembly. These high quality BAC sequences include 1,872 Phase-3 BAC regions (178.2 Mb) and 292 Phase-2 BAC regions containing at most one gap (20.8 Mb). Comparisons between WGS scaffolds and these BAC sequences showed that 89.7% of the selected BAC sequences were covered in ungapped alignments, with average sequence identity of 99.93%. The remaining 0.07% are due to single nucleotide differences. When gapped alignments are allowed, the total coverage of the BACs increased to 94.8%. This implied that we could use the additional 5.1% of the high quality BAC sequences (~10 Mb) to patch the gaps in the WGS scaffolds. Consequently, we incorporated all the high quality BAC sequences using the following approach: 5Kb sequences on the left and right end of each BAC or multi-BAC contig were extracted and searched against the Mt4.0 assembly. The sequence ranges in between the left and right flankers (in Mt4.0) were replaced by the corresponding sequence from the Mt3.5 BACs, thus effectively eliminating most of the sequence variants as well as the gaps between the two versions in the euchromatic regions.

### Medicago re-annotation overview

Our Medicago re-annotation strategy is a hybrid approach that attempts to combine a set of transcriptional and translational evidence. Mt3.5 legacy gene models, predictions from Augustus and FGENESH, and EST, 454 and RNA-seq expression data were combined using both EVidence Modeler (EVM) and MAKER with minor differences (Table 2). Precedence was given to the EVM predictions that were then supplemented with MAKER models that did not have a counterpart in the EVM dataset. Approximately 1,500 models for small secreted peptides predicted from custom HMMs [20] and community annotated genes were also added to generate the final Mt4.0 gene set. A schematic outline of the Medicago re-annotation pipeline is available in Figure 1B.

**Table 2:** Evidence tracks used in Medicago reannotation pipeline

**Type**	**Evidence**	**EVM**	**MAKER**
Prediction	AUGUSTUS	Yes	Yes
Prediction	FGENESH	Yes	Yes
Prediction	GENEMARK	No	Yes
Transcript	Medicago ESTs	Yes	Yes
Transcript	RNA-seq assembled with Rnnotator	Yes	Yes
Transcript	RNA-seq assembled with CLC	Yes	No
Transcript	RNA-seq assembled with CUFFLINKS	Yes	No
Transcript	Legacy Mt3.5 loci transferred using GMAP and liftOver	Yes	Yes
Protein	Plant uniref90 proteins	Yes	Yes
Protein	Six plant proteomes (*A. thaliana*, *G. max*, *P. trichocarpa*, *S. lycopersicum* and *O. sativa*)	Yes	Yes
Protein	GENEWISE with *A. thaliana*, *G. max* and *P.trichocarpa* proteins	Yes	No

### Training sets

**Figure 3:**
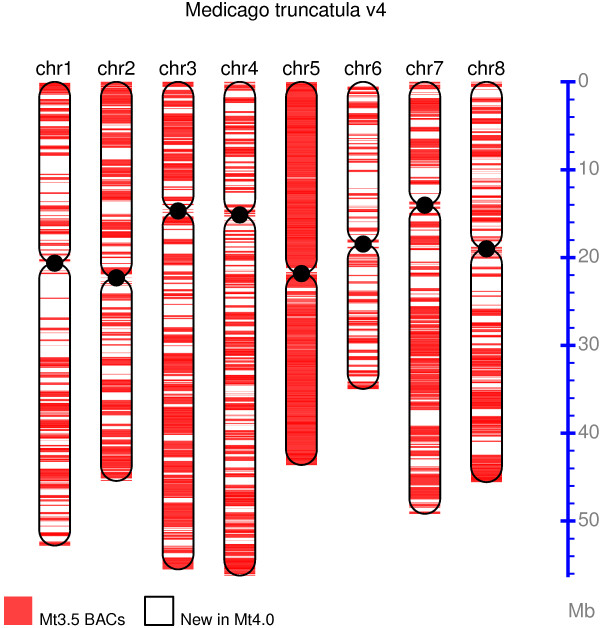
**Increased amount of chromosome-anchored sequences in Medicago Mt4.0 compared to Mt3.5.** Red-colored portion of the chromosomes represent BAC sequences used in Mt3.5, while the white regions on the chromosomes represent newly anchored sequences in Mt4.0.

For evaluation and optimization of our gene predictions, we developed manually curated training sets either using models with full-length transcript support (EST or RNA-seq) on chromosome 1 or genes directly transferred from Mt3.5 to Mt4.0 chromosome 5, which is the best-assembled chromosome in both Mt3.5 and Mt4.0 (Table 3 and Figure 3). We classified transcripts as ‘full-length’ using TargetIdentifier [21]. We used full-length transcripts (fl-ESTs and fl-Rnnotators) on chr1. We used ‘F’ class genes on chr5 which were considered the highest confidence class in Mt3.5 [3]. Both sets were manually verified by manual inspection using a genome browser, JBrowse [22].

**Table 3:** Statistics of the final assembly, including the total numbers of base pairs on each chromosome and unplaced scaffolds

**Seqid**	**Real**	**N’s**	**Total**	**% of real bases**	**% aligned to optical map**
chr1	50,275,726	2,715,429	52,991,155	94.9 %	86.9 %
chr2	43,694,219	2,035,453	45,729,672	95.5 %	84.3 %
chr3	52,386,245	3,128,907	55,515,152	94.4 %	83.8 %
chr4	54,533,855	2,048,528	56,582,383	96.4 %	89.6 %
chr5	43,376,507	254,224	43,630,731	99.4 %	92.6 %
chr6	31,992,419	3,283,294	35,275,713	90.7 %	79.3 %
chr7	46,512,325	2,660,098	49,172,423	94.6 %	85.4 %
chr8	43,183,948	2,386,037	45,569,985	94.8 %	81.9 %
chr total	365,955,244	18,511,970	384,467,214	95.2 %	85.7 %
Unplaced	24,050,008	4,319,556	28,369,564	84.8 %	n. a.

### Gene structure consolidations

Annotation data were consolidated using EVidence Modeler (EVM) [8] and MAKER [9]. We ran evidence modeler (EVM) using several sources of sequence and *ab initio* evidence. MAKER was run using a similar set of evidence (Table 2). We used an in-house tool GSAC (Genome Structure Annotation Comparison) to evaluate the performance of EVM and MAKER as well as the directly transferred predictions from Mt3.5 against our training sets. After several iterations of optimization via weighting parameter adjustment, EVM was found to be more accurate than either MAKER or Mt3.5 predictions and was therefore used as the main annotation pipeline. Mt3.5 models were used as one line of evidence for EVM, thus favoring the retention of these models when they agreed with EST/RNA-seq and protein alignment data.

The consolidated gene set consisted of the output of the EVM pipeline and gene predictions from the MAKER pipeline that did not intersect with these data sets, which were supplemented with the community contributed annotations. The community contributed annotations consisted primarily of small cysteine-rich peptides predicted by the SPADA pipeline [20], a small number of annotation updates contributed by community members and a small number of updates supported by proteomics data [7]. We manually removed overlapping models using our in-house editor, AnnotationStation. For tRNAs, we ran tRNAscan [23] and consolidated the output with existing Mt3.5 tRNAs. Final clean ups included removal of duplicate scaffolds, sequence contaminants (organelles and microbes, probably endophytic), and predictions less than 50aa in length, except for SPADA models that are known to be relatively short.

### Gene identifier assignments

Most of the gene identifiers (Medtr) have been preserved between Mt3.5 and Mt4.0. New identifiers have been instantiated to replace the gene identifiers previously found on the unanchored contigs. We have assigned gene identifiers based on the following three rules:

•Rule 1: All Medtr genes that can be moved over either in whole or in part will retain the same identifier;

•Rule 2: All contig genes (with identifiers like contig999_1) received new Medtr identifiers;

•Rule 3: New gene predictions in regions of the genome not present in Mt3.5 were assigned Medtr identifiers consistent with their chromosomal location.

When multiple old identifiers mapped to the same locus on Mt4.0 (e.g. when a new gene was a fused model), we used the EMBOSS ‘needle’ program [24] to select the legacy identifier with highest identity and coverage to carry over.

Newly instantiated genes inserted into gaps were assigned identifiers that maintained the monotonic sequence of identifiers, making use of sets of identifiers that had been reserved for each of the gaps based upon approximated size in the Mt3.5 pseudomolecules. Depending on the number of reserved identifiers for a particular gap, strides of 2, 3, 5 or 10 were selected to account for future genes. For example, to insert 2 genes between and Medtr1g009000 and Medtr1g009050, we added Medtr1g009010 and Medtr1g009020, using a stride of 10 in this case. However, when insufficient identifiers were available, we made use of the first digit of the identifier and inserted a “4” as in MedtrXg**4**XXXXX. For example, to insert 20 genes between Medtr1g009000 and Medtr1g009010, we would have used Medtr1g409010, Medtr1g409020 and so on. Consequently, the new identifier scheme still provides useful information about a gene’s chromosomal location and its neighbors.

### Functional annotation

Functional assignments were based on a weighted keyword (WK) approach (Hoover et al. in preparation). Briefly, each predicted protein was searched against a collection of databases (Priam, Uniref100, PFAM/TIGRFAM, CAZY, CDD) and motif finders (TMHMM, InterPro). Meaningful keywords were extracted from the definition lines of sets of best matches from each database. A set of heuristic rules were then used to score each candidate definition line and the highest scoring line was assigned to the query protein. A second iteration of the WK scoring algorithm was used to standardize functional assignments across members of paralogous gene families wherever possible. As a result of the protein naming pipeline, 37,561 genes (74%) contain informative protein names, while the remaining 13,367 genes are labeled as “hypothetical protein”.

### Repeat analysis and transposon classification

A multi-evidence approach was used to distinguish between canonical genes and transposon derived gene models. Gene predictions were classified as transposons-related based upon one or more of the following criteria: 1) intersection with computationally predicted repeats; 2) membership in a paralogous gene family composed predominantly of gene predictions that received a functional annotation of transposon from our naming pipeline; 3) matches to an in-house transposon protein database; 4) possession of an appropriate InterPro domain. This integrated repeat analysis pipeline improved classification of loci as either genes or transposable elements.

### UTRs and isoforms

Splice isoforms and UTRs were instantiated by running PASA [25] on Sanger/454 EST and RNA-seq data. Publicly available RNA-seq data (Additional file 1: Table S1) were assembled using a combination of *de novo* and genome-guided Trinity [26]. Transcript diversity was captured by assembling reads on a per tissue type and per time point basis pooling the biological replicates within each sample/treatment.

PASA was run twice to assemble the transcriptome data; first on EST data followed by UTR and isoform updates and then on the RNA-seq transcript assemblies. Within each gene locus, assemblies were filtered to remove any transcripts with low read depth, using RSEM [27]. Within the set of isoforms that were instantiated, we observed two different types of variation: within the UTR regions which did not affect the encoded protein sequence and within the coding regions which encoded variant proteins. Since RNA-seq reads from a wide variety of different tissue types (root, nodule, seedpod, blade and flower) were used in this high-throughput step of instantiating isoforms, without large-scale manual curation it is hard to verify the authenticity of all the computed variants. For example, within certain gene loci, the only variation observed was in the UTR regions, many of which showed only minute differences in the UTR start/stop positions.

To filter the excessive number of isoforms possibly due to read-mapping artifacts, we filtered through these isoforms using the following method: for every locus, identify sets of isoforms sharing the same coding region using the Gene Structure Annotation Comparison (GSAC) tool and retain only the longest transcript within each such set. A total of 6,377 gene loci (13% of all loci) contain more than one isoform. The most extreme case is gene locus Medtr8g070990 that encodes a putative RNA-binding protein and has 31 isoforms.

### Inferring synteny blocks derived from the papilionoid genome duplication event

To call synteny blocks, we performed all-against-all LAST [28] comparison of the predicted gene models of Medicago. We define syntenic blocks by chaining LAST hits with a distance cutoff of 20 genes, also requiring at least 5 gene pairs per synteny block. The collection of synteny blocks were further filtered through “1:1” syntenic depth constraint using QUOTA-ALIGN [29]. QUOTA-ALIGN identifies the best scoring set of blocks while subject to the constraints that no block should overlap another block either vertically or horizontally on the dot plot. This block-level filtering step removed low-scoring blocks due to computational artifacts and older duplication events [29].

### The Mt4.0 release

The sequence data are released as a set of Mt4.0 pseudomolecules comprising FASTA files and their alignments to the genetic and optical maps. Annotations of genes, TEs, tRNAs are available as GFF files and as CDS and protein sequences in FASTA format. We also generated a “chain” file (coordinate mapping between Mt3.5 and Mt4.0) that can be used in conjunction with the UCSC liftOver tool, in order to quickly map any genomic features or annotations from the Mt3.5 assembly to the Mt4.0 assembly. The release files are available at the JCVI Medicago website (http://www.jcvi.org/medicago). The same set of Mt4.0 assembly and associated gene models are also available in GenBank under accession **APNO00000000**. The optical maps for all 8 chromosomes are available in the Genbank nucleotide database under accessions **MAP**_**000013** to **MAP**_**000020**.

## Results and discussion

### Assembly completeness

We report a much-improved Medicago v4 assembly release (Mt4.0). Mt4.0 pseudomolecules are based upon a new whole genome assembly that also incorporates sequences from the BAC-based Mt3.5 assembly wherever possible. Mt4.0 release included substantially more Illumina whole genome shotgun sequences to increase depth. The new assembly has now placed most of the previously unanchored sequences onto the chromosomes. Mt4.0 spans 384.5 Mb containing 360.0 Mb of real bases of which ~86% are aligned to the optical map. There are also unanchored scaffolds that span 28.4 Mb. This is a dramatic improvement over Mt3.5 which was composed of pseudomolecules spanning 297.1 Mb with 245.3 Mb of real bases, 31.8 Mb of unanchored BAC contigs containing 17.6 Mb of real bases plus 104.2 Mb of relatively short contigs derived from Illumina WGS sequencing [3]. The improvement of completeness over previous version is large and apparent on all 8 chromosomes (Figure 3). Chromosome 5 contains the least amount of newly anchored sequences, consistent with the fact that it was already the best assembled chromosome in Mt3.5 [3].

We also determined the completeness of the Mt4.0 assembly using CEGMA [30], which identifies the presence of 248 conserved eukaryotic genes. A total of 234 (94%) ultra-conserved CEGs are present in the genome as complete gene models, and 243 (98%) CEGs are present including partial gene models. This is an improvement over the Mt3.5 assembly, which contained only 88% and 97% of the CEGs as complete and partial, respectively. Using a Medicago unigene set (combining the DFCI Medicago Gene Index and the PlantGDB putative transcript assemblies, a total of 87,639 sequences) as an independent metric of completeness, a total of 92.3% of the unigenes can be mapped to Mt4.0 with ≥ 90% identity and ≥ 50% coverage, which is comparable to the level in Mt3.5 [3].

### Assembly validation through optical and genetic maps

Approximately 329 Mb of Mt4.0 sequences were aligned to the optical map, which is a significant improvement in comparison to Mt3.5, that only has ~203 Mb of sequences aligned to the optical map. The length statistics and evaluation of completeness against optical map alignment per chromosome is available in Table 3.

**Figure 4:**
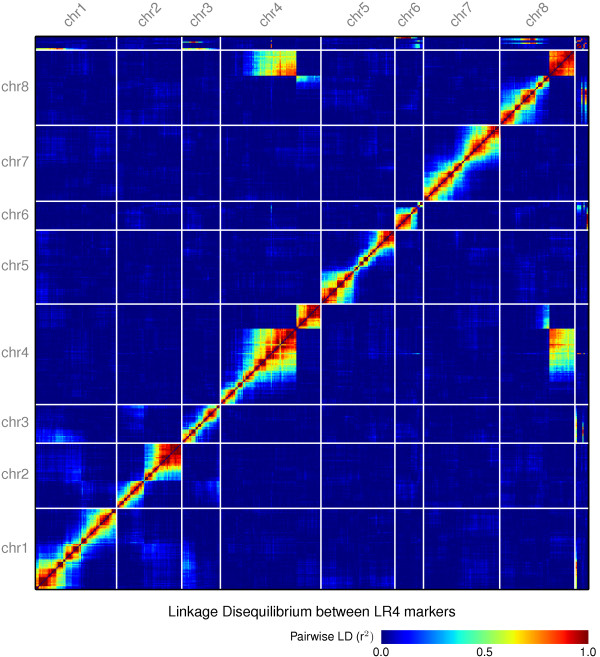
**Heatmap of linkage disequilibrium between pairwise SNP markers in the Mt4.0 assemblies.** Pairwise linkage disequilibrium (LD) between markers was calculated as *r*^*2*^ value based on segregations of individuals within LR4 mapping population.

A *M. truncatula* RIL population LR4 (DZA315.16 × Jemalong.J6) mapping population was genotyped to construct a high density genetic map with 12,287 markers (12,002 SNP markers and 285 SSR markers) on 139 RIL individuals. Jemalong J6 is a cultivar that is very close to the reference strain A17, therefore most of the polymorphic sites are from the other parent DZA315.16. The heat map of pairwise LD revealed that most of the assembled chromosomal sequence pseudomolecules are consistent with the genetic map in that the extent of most linkage appears close to diagonals (Figure 4).

Comparison between the genetic and optical maps revealed a major structural incongruity between the LR4 genetic map and the reference cultivar A17. The LR4 mapping population apparently shows a genetic linkage between the lower arms of chromosomes 4 and 8 (Figure 4). This discrepancy appears to be due to a reciprocal translocation between chr4 and chr8 in the lineage of A17 [31], but is absent from the parents of the LR4 population (J6 x DZA315.16) [13,32]. This reciprocal translocation is further supported by optical mapping and the A17 genetic map (data not shown).

Together, the optical map and high-density genetic map are responsible for anchoring ~93% of the sequences onto the 8 Mt4.0 chromosomal sequence pseudomolecules. In addition, the two maps were capable of identifying and correcting 9 mis-joins from ALLPATHS-LG due to repetitive sequences (Figure 2). The combination of the two maps allows sequences to be anchored with much higher confidence than using either map alone.

### Confidence of gene calls

Gene predictions were classified into levels of confidence based on the extent and quality of their alignments to transcripts, proteins, and genome alignments as described in Additional file 1: Table S2. The alignment criteria were selected based on frequency distributions of sequence identity and coverage, as well as visual proofing via JBrowse. The characteristics of high-confidence and low-confidence genes are distinctly different. The mean size of high-confidence genes is 3,280 bp, more than doubled compared with 1,526 bp for low-confidence genes. The high-confidence genes have an average of 5.5 exons, again double when compared with 2.7 exons per low-confidence gene (Table 4). The shorter gene length, combined with the observation that very few alternative splicing transcripts were identified in the low-confidence gene set (Table 4), suggested that some low-confidence genes may be potential gene fragments that often resulted from transposable element activity [33,34].

**Table 4:** Characteristics of high confidence and low confidence gene sets

	**High confidence (HC)**	**Low confidence (LC)**
Number of genes	31,661	19,233
Number of single-exon genes	6,103 (19%)	5,351 (28%)
Number of multi-exon genes	25,558 (81%)	13,882 (72%)
Number of genes with alternative transcript variants	6,041 (19%)	347 (2%)
Number of predicted transcripts	42,481	19,838
Number of distinct exons	174,533	52,850
Mean gene locus size (first to last exon)	3,280	1,526
Mean transcript size (UTR, CDS)	1,618	841
Mean number of transcripts per gene	1.3	1.0
Mean number of distinct exons per gene	5.5	2.7
Mean exon size	308	296

### Comparison of Mt4.0 annotation with prior version

As published, Mt3.5 (with the last annotation release Mt3.5v5) contained 62,379 annotated gene loci with 14,309 additional predictions classified as transposable element-related. Mt3.5 genes were curated by the IMGAG consortium using the EuGene pipeline [35]. For Mt4.0, all annotation work was carried out at JCVI. Thus we used both a different annotation pipeline and a different confidence classification system. This resulted in the prediction of 31,661 high confidence genes, 19,233 low confidence genes (total 50,894) together with 16,504 predictions classified as TE-related. This set of high and low confidence genes represents the official release of Mt4.0 annotation set. A further set of 19,229 unsupported predictions that are only *in silico* predictions with minimal support from databases or other species are also available for download on the JCVI Medicago website, providing the most exhaustive set of gene loci that we have predicted.

In tracking the continuity of genome releases, 82% of the ~62,000 genes annotated in Mt3.5 are captured in the current set of high and low confidence predictions with another 14% now classified as unsupported. The remaining 4% of Mt3.5 genes have no counterpart in the Mt4.0 release. Most Mt3.5 genes (74%) are unchanged or found with only minor changes in structure in Mt4.0; 20% are involved in a merging of two loci (70% of merged loci originated from unanchored contigs, which were likely partial gene models in Mt3.5); splits and more complex associations account for the remaining 6% of Mt3.5 gene loci.

We have tracked identifiers (e.g. Medtr1g010100) between Mt3.5 and Mt4.0 and retained them, wherever possible. We have assigned new identifiers to the newly instantiated genes (including those previously found on the unanchored BACs and Illumina contigs) using identifiers reserved for the gaps in the previous Mt3.5 pseudomolecules. Because of some inversions or rearrangements in Mt4.0 vs Mt3.5, the order of loci down the pseudomolecules is not strictly monotonic. Additionally, since there were a few regions where insufficient identifiers had been set aside to accommodate all the new genes in a gap, we made use of the leading digit in the six-digit identifier string to provide unique loci that still preserved information about their location on the pseudomolecule. We note that all gene identifiers are unique in the Mt3.5/Mt4.0 identifier space. Identifiers that are retired are never re-used. Overall, approximately 60% of the Medtr identifiers in Mt4.0 are directly inherited from Mt3.5. Most of the remaining Mt4.0 identifiers are assigned to genes previously present on BACs or Illumina WGS contigs. A small number are new assignments due to gene splits (724) or merges (2,331) following the Arabidopsis nomenclature guidelines or to movement of a sequence region (and its associated genes) onto another part of the genome during Mt4.0 construction. A full look-up table between Mt3.5 loci and Mt4.0 loci is provided on the JCVI Medicago website.

### Mt4.0 as an improved reference for legume comparative genomics

A whole genome duplication (WGD) event occurred in the common lineage of papilionoid legumes [3,36,37]. With the incomplete genome assembly of Mt3.5, the residual signature of papilionoid duplication event was evident but much weaker than that detected in the soybean genome. The average number of homologous gene pairs per block was a striking ~2.5x fold lower than that in soybean [3].

**Figure 5:**
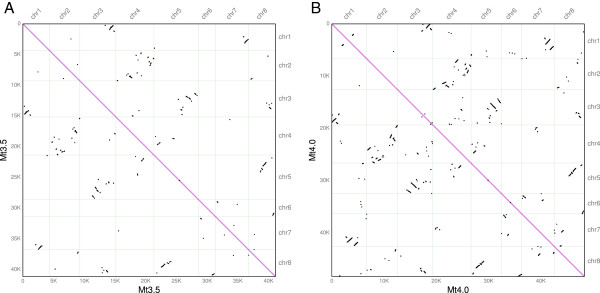
**Syntenic dot plot of Medicago genome versus itself, showing blocks derived from papilionoid whole genome duplication event.** Contrasting **(A)** Mt3.5 and **(B)** Mt4.0 with the same synteny block chaining settings (see Methods).

With the new Mt4.0 assembly, the number of retained gene duplicates that can be detected has increased significantly. In Mt3.5, a total of 109 blocks containing 1,628 gene pairs were found to be involved in papilionoid WGD event, with an average size of 15 gene pairs. By comparison, a similar analysis within Mt4.0 identified a total of 186 blocks containing 4,522 gene pairs that originated from the WGD event, with an average block size of 24 gene pairs. The largest WGD block in Mt4.0 contains a total of 232 gene pairs, which has increased substantially from the 62 gene pairs in Mt3.5. The nearly ~3x fold increase in retained WGD duplicates and the increased synteny block sizes that can be detected are due to the substantial improvement of contiguity in Mt4.0 assembly, and effectively explains the previously claimed discrepancy between Medicago and soybean on the papilionoid WGD event. In addition, the apparent lack of major duplication blocks in Mt3.5 (Figure 5A) had led to the speculation that there was likely a period of extensive rearrangements after the duplication event [38]. We argue that the rate of genome rearrangements may be over-estimated. Indeed, we found that the new Mt4.0 release significantly improved detectability of legume-wide whole genome duplication event (Figure 5B). Nine major duplication blocks become evident from the self-comparisons in Mt4.0, involving chromosome pairs of chr1-chr3, chr1-chr7, chr2-chr4, chr3-chr5, chr3-chr8, chr4-chr5, chr5-chr8, ch6-chr7, chr6-chr8, which involves every single chromosome of Medicago (Figure 5B).

**Figure 6:**
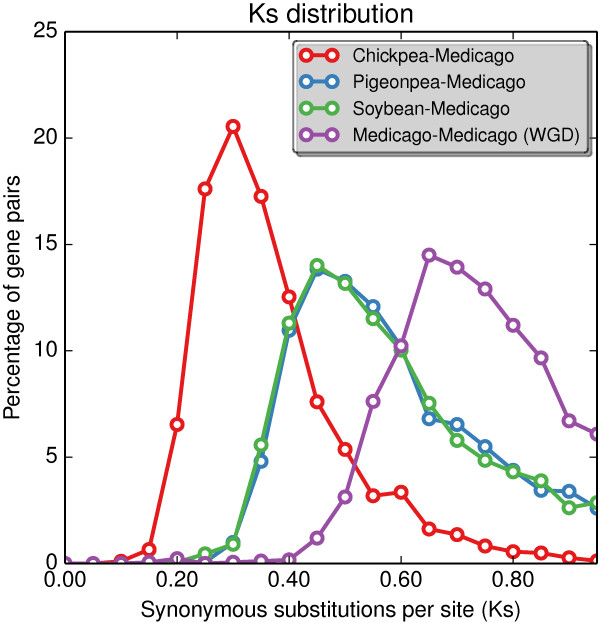
***Ks *analyses of comparisons between legume species with whole genome sequences.** Percentage of gene pairs is taken as the counts of syntenic homologs within a *Ks* range (with bin sizes of 0.05) divided by all the syntenic homologs identified.

Comparisons between Medicago and other sequenced legume genomes reveal better separation of the speciation and WGD events (Figure 6). The papilionoid WGD event has a *Ks* mode of 0.64, more ancient than the divergence to pigeonpea and soybean with *Ks* modal values of 0.46 and 0.42, respectively. Among the selected legumes, chickpea is the closest to Medicago at *Ks* of 0.28. This is consistent with the legume phylogeny since chickpea and Medicago both belong to the galegoid clade, while pigeonpea and soybean belong to the millettioid clade [38,39]. The improved set of Medicago gene models in Mt4.0 will continue to serve as a great resource for comparative genomics across legumes.

### JCVI Medicago website

**Figure 7:**
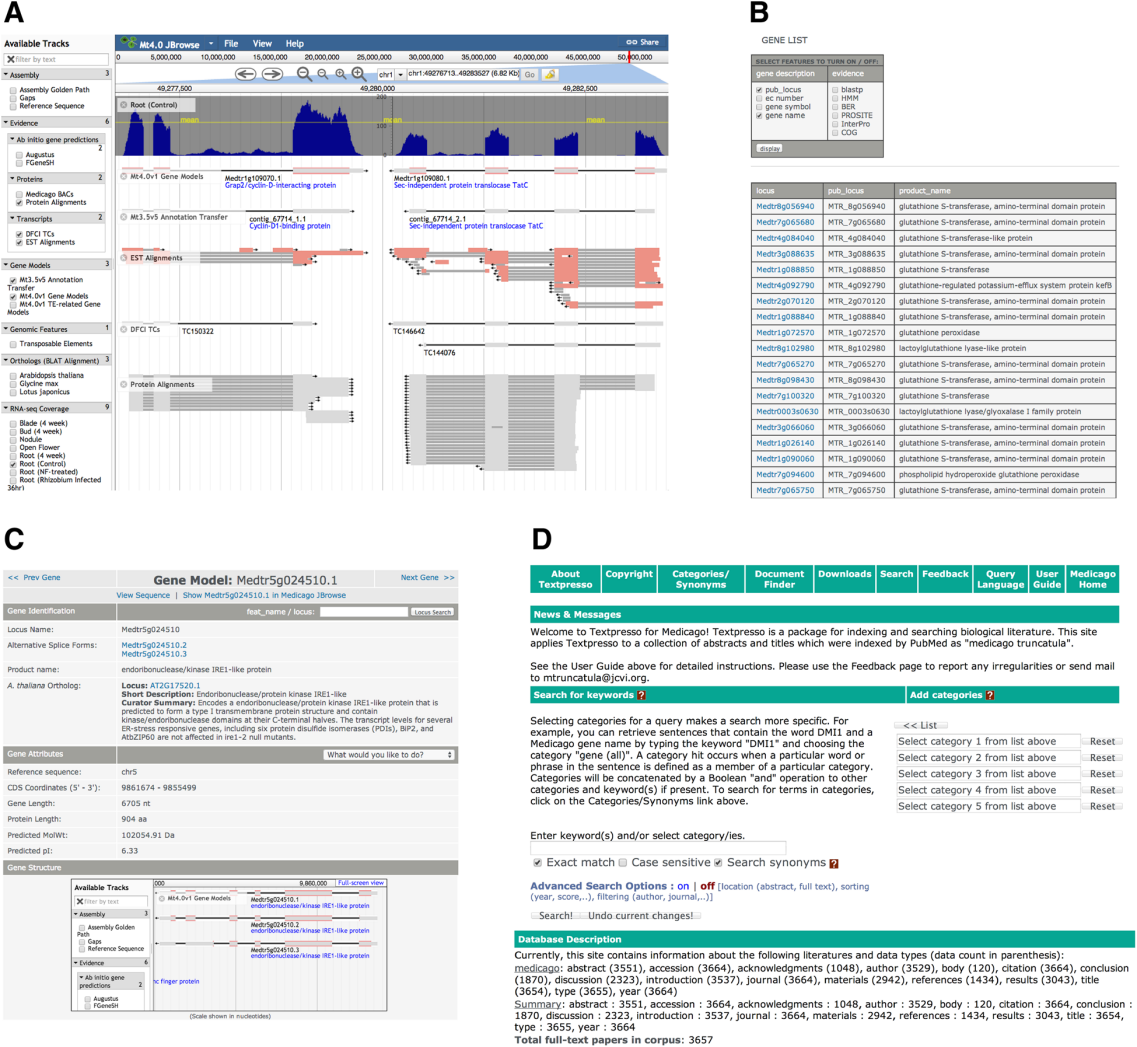
JCVI website for Medicago genome resources, showing a number of services and tools to interact with the Mt4.0 datasets (A) JBrowse shows the alignment of annotation evidence to the genome; (B) Keyword search supports extraction of gene lists; (C) TAIR-style gene information page of Mt4.0 gene models; (D) Textpresso for mining Medicago-related literature.

The JCVI Medicago website (http://www.jcvi.org/medicago) has been updated with the Mt4.0 data and contains a number of bioinformatics utilities to search against the Medicago database. Five major services and resources are offered on the website: 1) BLAST service that allow searches against the genome and the proteome; 2) Genome browsers that allow interactive navigation of the genome, through both JBrowse and the previously deployed GBrowse; 3) Keyword and locus search for your favorite genes; 4) Gene information page that provide detailed information including functional searches and domain structures about every predicted gene locus; 5) Textpresso that provides access to related Medicago literature. The web interface for a selected set of tools is shown in Figure 7.

In addition to the query functionalities, we have also instantiated a Community Annotation Portal that extends the functionality of the original rice EuCAP [40] and also supports mutant and allele information previously developed by Frugoli et al. [41]. This allows researchers to edit gene structure, gene function and add mutant information in a user friendly interface. Researchers can become a *“*community annotator” and be able to edit gene function, gene symbol, associated publications and GenBank identifiers, assign mutant information, alleles and phenotype for any given genes. Through this interface, community members can provide their expertise to annotate or endorse their favorite genes and gene families as a complementary and long-term solution to our continuing Medicago genome curation efforts.

### Future plans

The ultimate goal of genome curation is to produce a gap-free genome [42]. Although the Mt4.0 release represents our best efforts so far, there are still gaps in the assembly as well as unanchored scaffolds that have not yet been incorporated into the pseudomolecules. We will obtain higher resolution GBS map from more individuals. We are planning more mate pairs to anchor currently unplaced scaffolds, as well as PacBio sequences to close gaps. We expect to uncover or be informed of errors and omissions in these sequences, and implement the corrections in the Mt5.0 release.

## Conclusion

We describe a new *Medicago truncatula* genome release Mt4.0, representing substantial improvements over the previous Mt3.5 which was published in Nature in 2011. The Mt4.0 assembly now has ~93% of the sequences anchored onto the chromosomes (compared to 71 % in the previous release) and has been carefully validated against the optical map as well as a high-density genetic map. The heavily curated chromosomal sequences and associated gene models will serve as a much better reference for legume biologists and plant physiologists. We have documented several informatics challenges during the curation of Medicago genome and presented our solutions to those challenges. For example, in order to maximally preserve compatibility with legacy Mt3.5 gene naming, we implemented rules to insert new identifiers and have provided detailed tracking of each gene in Mt3.5. The techniques we used are of special interest to researchers who are also ‘upgrading’ their reference assemblies and annotations. Such genome upgrading is getting more popular in recent years due to the drop in sequencing cost. We further report the associated web-accessible resources that we have built around the Medicago genome releases. We host databases, genome browsers, searching utilities and community annotation services on our JCVI Medicago web server.

### Availability of supporting data

The Mt4.0 assembly and associated gene models are available in GenBank under accession **APNO00000000**. The optical maps are available in the Genbank nucleotide database under accessions **MAP_000013** to **MAP_000020**. Supplementary tables are included as an additional file:Additional file 1: Table S1. Available RNA-seq data used in Mt4.0 for UTR and isoform instantiation. Additional file 1: Table S2. Classification of genes into high and low confidence classes.

## Competing interests

The authors declare that they have no competing interests.

## Authors’ contributions

HT, VK, SB, BR, AC, and CT compiled and curated the Mt4.0 genome release. SZ and DS performed the optical map alignment. LG provided the LR4 mapping population and helped to construct the genetic map. KC and MY carried out the MAKER gene annotation. HG and KM carried out the repeat analysis and transposon classification. HT, BR and CT drafted the manuscript. CT conceived the study and participated in its design. All authors read and approved the final manuscript.

## Supplementary Material

Additional file 1: Table S1 Available RNA-seq data used in Mt4.0 for UTR and isoform instantiation. **Table S2.** Classification of genes into high and low confidence classes. Click here for file
