# Supplementary material for: An improved genome release (version Mt4.0) for the model legume Medicago truncatula
Source: BMC Genomics. 2014 Apr 27;15:312. doi: 10.1186/1471-2164-15-312 (PMC4234490; doi:10.1186/1471-2164-15-312)
Supplement: Additional file 1: Table S1 — Available RNA-seq data used in Mt4.0 for UTR and isoform instantiation. Table S2. Classification of genes into high and low confidence classes. [file 1471-2164-15-312-S1.docx]

**Additional file 1**

**SI Table 1.** Available RNA-seq data used in Mt4.0 for UTR and isoform instantiation.

| **Source** | **Sequencing library** | **Tissue** | **Treatment** |
| --- | --- | --- | --- |
| INRA | Paired-end 54bp | Nodule | Control |
| UW-Madison [SRP018396] [1] | Single-end 100bp | Root | Nod-factor treated; Control |
| J. Mun [2] | Single-end 101bp | Root | Rhizobium infected; Control |

**SI Table 2.** Classification of genes into high and low confidence classes.

|  | **Identity** | **Coverage** |
| --- | --- | --- |
| **High confidence (31,661)** | | |
| BLASTN to transcripts | ≥ 97% | ≥ 30% |
| BLASTP to 10 plant proteomes | ≥ 70% | ≥ 70% |
| **Low confidence (19,233)** | | |
| BLASTN to transcripts | ≥ 97% | 10% ≤ cov < 30% |
| BLASTP to 10 plant proteomes | ≥ 30% | 30% ≤ cov < 70% |
| LAST to 10 plant genomes | Not enforced | ≥ 30% |

**References**

1. Volkening JD, Bailey DJ, Rose CM, Grimsrud PA, Howes-Podoll M, Venkateshwaran M, Westphall MS, Ane JM, Coon JJ, Sussman MR: **A proteogenomic survey of the Medicago truncatula genome**. *Molecular & cellular proteomics : MCP* 2012, **11**(10):933-944.

2. Laporte P, Lepage A, Fournier J, Catrice O, Moreau S, Jardinaud MF, Mun JH, Larrainzar E, Cook DR, Gamas P *et al*: **The CCAAT box-binding transcription factor NF-YA1 controls rhizobial infection**. *Journal of experimental botany* 2014, **65**(2):481-494.
